# Brain fatigue in Graves’ disease: symptoms and presentation of a possible mechanism at the cellular level

**DOI:** 10.1530/ETJ-25-0172

**Published:** 2026-03-05

**Authors:** Karin Tammelin, Mats Holmberg, Agneta Lindo, Birgitta Johansson, Lars Rönnbäck, Helena Filipsson Nyström

**Affiliations:** ^1^ Department of Endocrinology, Sahlgrenska University Hospital, Gothenburg, Sweden; ^2^ Institute of Medicine, Sahlgrenska Academy, University of Gothenburg, Gothenburg, Sweden; ^3^ Wallenberg Center for Molecular and Translational Medicine, Västra Götaland Region, Gothenburg, Sweden; ^4^ ANOVA, Karolinska University Hospital, Stockholm, Sweden; ^5^ Department of Medicine, Huddinge, Karolinska Institutet, Stockholm, Sweden; ^6^ Institute of Neuroscience and Physiology, Department of Clinical Neuroscience, Sahlgrenska Academy, University of Gothenburg, Gothenburg, Sweden

**Keywords:** Graves’ disease, fatigue, brain fatigue syndrome, astrocyte, autoimmunity, CNS inflammation

## Abstract

Fatigue in patients with Graves’ disease (GD) is characterized by a profound lack of mental energy that affects daily functioning, including work. This symptom is particularly prominent in the early stages of the disease, affecting more than 50% of patients, but in some cases, it persists even after successful endocrinological treatment and restoration of euthyroidism. Individuals with persistent fatigue often seek support. Because this tiredness originates in the brain, we refer to it as brain fatigue. It is accompanied by a cluster of interconnected symptoms as cognitive, sensory, and emotional, which we define collectively as brain fatigue syndrome (BFS). BFS is marked by reduced perceived energy levels and associated impairments across multiple domains. The aim of this paper is to improve the understanding and identification of BFS in GD and to propose potential treatment options. We also propose a hypothesis, supported by robust preclinical evidence, that the inflammatory response in this autoimmune disorder may lead to astrocyte dysfunction, impairing neuronal signaling for multiple neurotransmitters. This could reduce the efficiency of brain information processing, increase activation of larger brain areas, and diminish glucose uptake from the bloodstream. Such changes may result in widespread brain dysfunction, culminating in an energy crisis that manifests as profound fatigue and cognitive, sensory, and emotional impairments. However, this hypothesis needs to be tested in humans, particularly regarding the persistence of brain fatigue in GD after normalization of thyroid hormone levels.

## Introduction

Fatigue beyond normal is a common symptom in patients diagnosed with Graves’ disease (diffuse autoimmune hyperthyroidism; GD) both before and, for some people, even after medical treatment (1). This fatigue is markedly different from ordinary tiredness or sleepiness in healthy individuals. It does not resolve with rest or sleep and may persist for extended periods. There is a significant risk that this fatigue becomes chronic and continues to impair daily functioning long after the initial onset. Limited endurance is further reduced during sensory stimulation and when cognitive tasks are performed for extended periods. Normal stimulation situations, such as reading for meaning, participating in conversations, or preparing a dinner, can be perceived as exhausting. The ability to work or participate in ordinary activities is reduced as all impressions and information are perceived as overwhelming. Another typical feature is a disproportionally long recovery time needed to restore energy.

Long-lasting fatigue with cognitive and emotional symptoms has previously been highlighted in both hypothyroidism (2) and GD (1, 3). Similar long-term problems after treatment for GD are reported in several studies, although not defined as fatigue (4, 5). Both hypothyroid patients and those with GD report fatigue with limited endurance as one of the most troublesome symptoms, resulting in difficulty taking part in everyday activities combined with reduced quality of life (QoL) and work ability (3, 4, 6, 7). Fatigue is distinguishable from psychiatric diseases and can occur both with and without depression or anxiety (1).

Cognitive symptoms are commonly reported by patients with GD suffering from fatigue but are seldom captured with standard neuropsychological tests (1, 8, 9), although a slight difference has been reported when using a more demanding cognitive test (10). However, subjective cognitive problems, such as concentration difficulties, memory problems, and slowness in thinking, are prevalent in patients with GD (1, 4, 11).

Current knowledge in neuropsychiatry, neuroimaging, and neurobiology related to autoimmune thyroid disease has recently been reviewed (12). Reduced QoL, cognitive difficulties, depression, and anxiety are commonly reported, and these symptoms are believed to result from thyroid dysfunction, metabolic disturbances, dysregulated autoimmune activity, and altered connectivity between brain regions. It is also well established that debilitating fatigue is a significant long-term issue in various neurological conditions, including acquired brain injuries (13, 14), autoimmune systemic inflammatory diseases, and endocrine disorders (1, 15), all of which affect the central nervous system (CNS) either directly or indirectly. Zielinski (2019) concluded that autoimmune diseases can induce brain inflammation and disrupt neuronal network connectivity, contributing to fatigue (16).

The aim of this paper is to demonstrate the brain fatigue syndrome (BFS) in GD, propose potential treatment options, and present a theory regarding the underlying mechanisms that may cause significant disruption to daily life, even when thyroid hormone levels are within the normal range. Our goal is to make BFS, often characterized by persistent cognitive and emotional symptoms, identifiable and understandable to researchers, clinicians, and patients. Drawing on neurobiological data, particularly evidence of astrocyte dysfunction in CNS inflammation, we propose a hypothesis for the underlying mechanisms of this syndrome (17), with a specific focus on GD. We aim to identify gaps in current knowledge, highlight potential treatment strategies, and present ongoing studies in GD. Ultimately, we hope to inspire further research that will elucidate the origins of BFS and lead to effective treatments for affected patients.

## GD and fatigue

Only a limited number of articles report on fatigue in GD. In a study conducted by our group, 89% of women with severe newly diagnosed GD with hyperthyroidism suffered from brain fatigue (according to results from the Mental Fatigue Scale, MFS). After 15 months of treatment, this brain fatigue was still present in 38% of the patients (1). Among individuals with untreated GD, 56% reported fatigue when assessed using the Fatigue Severity Scale (FSS) (18). Similarly, long-term fatigue was reported by 53% of patients using the Nottingham Health Profile (3). In an 8-year follow-up of patients after GD, 25% reported that they were not fully recovered and the main symptom was fatigue (19). In a 2006 review (20), cognitive complaints were noted in 16–54% and fatigue in 24–95% of patients with untreated hyperthyroidism. In the same review, long-term cognitive complaints were reported in 35–41% and fatigue in 39–58%. Patients report a high rate of fatigue, and clinically, fatigue is well-known to endocrinologists. They found it difficult to handle due to a lack of knowledge regarding underlying mechanisms and treatment options. In addition, there is no consensus on how to evaluate treatment efforts and there is no agreement on its terminology. Terms such as ‘fatigue’, ‘mental fatigue’, ‘cognitive symptoms’, or ‘brain fog’ have all been suggested in thyroid diseases (1, 2, 21, 22, 23).

### Typical symptoms of brain fatigue in GD

We use the term BFS. To date, only the FSS, MFS (1, 18), and fatigue components embedded in QoL questionnaires (3) have been reported in the context of GD. There is currently no consensus on which fatigue scale should be used for GD, nor is there a universally accepted definition of fatigue. This lack of agreement contributes to the complexity and elusiveness of fatigue as a clinical and research concept.

Our definition of BFS includes cognitive, sensory, and emotional symptoms, with central features being profound fatigue and a lack of mental energy. These symptoms (typical and associated) are closely interconnected. In patients with GD, these symptoms may coexist alongside the typical signs of hyperthyroidism. Fatigue can also be persistent in some individuals, potentially explaining prolonged reductions in QoL (7) and diminished work ability (3).

### The brain fatigue syndrome

#### Typical symptoms

- An unusual drain of mental energy upon mental activity.
- Impaired attention and concentration over time.
- Following over-exertion, a long recovery time disproportionate to the exertion level.
- Diurnal variation of the fatigue symptom with the fatigue often being better in the mornings and worse in the afternoons and evenings, often with variations from one day to the next.

#### Associated symptoms

- Memory problems.
- Slowness of thinking.
- Reduced initiative.
- Increased tendency to become emotional/tearfulness.
- Irritability.
- Stress sensitivity.
- Sleep problems.
- Sensitivity to or intolerance of light and noise.
- Headaches following over-exertion.

More than 70 fatigue assessment scales exist either tailored to specific disorders or designed for general use (24). In addition, quality-of-health scales are used, including subscales, as the Thyroid-Specific Patient-Reported Outcome (ThyPRO) questionnaire, which includes subscales that allow for the evaluation of cognitive complaints and fatigue (25). In our studies, we have employed the MFS (26), developed from extensive research and clinical experience across various diagnostic groups, including neurological diseases, acquired brain injuries, and endocrine disorders (14, 15, 27, 28, 29, 30). The MFS encompasses symptoms of brain fatigue, cognitive difficulties, emotional lability, sensory hypersensitivity, and sleep disturbances. A total score of ≥10.5 on the MFS indicates the presence of BFS (26, 28, 31). The MFS has been validated for use in patients with stroke and traumatic brain injury, both of which frequently involve persistent fatigue. The symptoms included in the MFS are derived from those commonly reported by neurological patients (14) confirmed through structured interviews to ensure item validity. The total MFS score correlates with cognitive processing speed and work ability. Furthermore, individual MFS items show internal consistency and represent a distinct construct, separate from depression and anxiety (26). Although the MFS has not yet been validated for GD, our research and clinical experience suggests that GD patients suffering from fatigue identify strongly with the symptoms described in the MFS and report experiencing debilitating fatigue (1).

## Thyroid immunology and the central nervous system

Cognitive and mood-related psychiatric symptoms are common in both untreated hypo- and hyperthyroidism (32), but it is unclear why some patients do not recover fully when euthyroidism is restored. A possibility is a lasting effect from the underlying immunological mechanisms (16). We have previously shown that the hippocampus and amygdala are smaller in hyperthyroidism with recovery in euthyroidism (33), proving that GD affects the volume of brain structures. Functional effects on neural networks have also been detected in patients with hyperthyroidism relative to healthy controls (34, 35, 36). In GD, thyroid autoimmunity also results in a systemic inflammatory response. GD is caused by a loss of self-tolerance to thyroid antigens and activation of CD4+ T lymphocytes to primarily type 1, but also type 2 helper T cells, and interleukin (IL)-17-producing T-helper lymphocytes. There is production of pro-inflammatory cytokines (37, 38, 39, 40, 41, 42), including interferon-γ, IL-6, tumor necrosis factor (TNF)-α, and IL-17, and chemokines attracting lymphocytes into the thyroid. This results in activation of B cells and production of TSH receptor antibodies (TRAbs), of which some are stimulatory and causes hyperthyroidism and goiter (38). In Graves’ orbitopathy, TRAbs activate both retro-orbital TSH receptors and insulin-like growth factor I receptors, causing cell proliferation and inflammation. Antibodies targeting thyroid peroxidase (TPOAb) and thyroglobulin (TgAb) are also frequent.

The classical thyroid antibodies (TPOAb, TgAb, and TRAb) bind not only to antigens in the thyroid gland but also to antigens in other tissues. TSH receptors are expressed in the hippocampus and on axons of cortical neurons located on astrocytes and in vascular endothelium (43, 44). They are also found on microglia (44), where TRAb stimulation *in vitro* leads to activation and pro-inflammatory cytokine release (45). TPOAbs bind to a subpopulation of astrocytes (46). TgAb binds to cerebral vascular endothelial cells (47), but there is also evidence of cross-reactivity with acetylcholinesterase in GD (48, 49).

The TSH receptor belongs to the large family of G-protein-coupled receptors. These transmembrane receptors are vital in many physiological processes, including immune response and hormone signaling, but are also frequent targets for autoantibodies (50). Antibodies to other G-protein-coupled receptors, besides TRAb, are common in GD (51, 52), and we and others have shown a broad response in beta-1 adrenergic receptor antibodies as well as of antibodies targeting muscarinic type 2 receptors and angiotensin II type 1 receptors (51, 52), with a reduction along with antithyroid treatment of the two former antibody titers (52).

Besides the possibility of mental consequences in GD being caused by antibodies, the cytokine response is necessary to spread the immunological processes to the CNS. IL-6, TNF-α, and IL-17, which are all elevated in serum of patients with GD (40, 41), are possible mediators of blood–brain barrier (BBB) disruption (53, 54). Evidence points to the IFN-γ-driven, kynurenine pathway as the link between systemic inflammation and mental consequences (55, 56). This immunological pathway is systemically activated in GD (37, 39).

We propose that thyroid autoimmunity causes an activation of inflammatory pathways, whereby inflammatory mediators cross the BBB barrier, activate astrocytes, and cause dysfunction of astroglial support during glutamate transmission (57). Systemic inflammation affects the BBB from the ‘blood side’ (54, 58) and affects endothelial cells and pericytes as well as astrocyte progenitors, which surround the small blood vessels and then form extensive cell networks inside the brain through gap junctions.

In the rare Hashimoto’s encephalopathy, associated with autoimmune thyroid disease, evidence suggests that intrathecal inflammation is started by thyroid antibodies crossing the BBB (46, 59). Thyroid antibodies are then believed to react with some of the many antigens that share homology with thyroid autoantigens. This might lead to epitope spreading (60), which could be the case in GD. The mental consequences of immune system activation, such as cognitive difficulties, fatigue, depression, and anxiety, are described in other conditions, as antibodies and pro-inflammatory cytokines can be produced in the CNS or reach the brain through the BBB disrupted by the inflammatory response (54, 58). To discuss possible implications of this, we first need to briefly describe the role of astrocytes.

## About BFS – symptoms and hypotheses regarding cause

BFS is characterized by a lack of brain energy, and we propose disturbances in neurotransmission and reduced efficiency in the brain’s information processing (17). Several hypotheses have been suggested to explain the underlying mechanisms of this fatigue. Impaired dopamine signaling in the frontal regions of the brain has been considered particularly important (61). Our studies with methylphenidate, which increases dopamine and noradrenaline levels in these regions, demonstrate improvements in concentration and attention among affected individuals (62). However, stamina and work capacity do not appear to improve, suggesting that additional signaling systems beyond dopamine and noradrenaline are involved. Serotonin likely plays a role, as emotional symptoms, such as tearfulness and irritability, are alleviated by low doses of SSRIs, which increase serotonin levels (63).

A unifying explanation may involve impaired glutamate signaling. This could account for insufficient energy supply to the brain, reduced transmission across multiple signaling systems, and nonspecific activation of larger neuronal circuits, some entirely unrelated to the task at hand, resulting in inefficient information processing (17). It is important to note that a healthy brain operates with remarkable energy efficiency.

**Figure 1:**
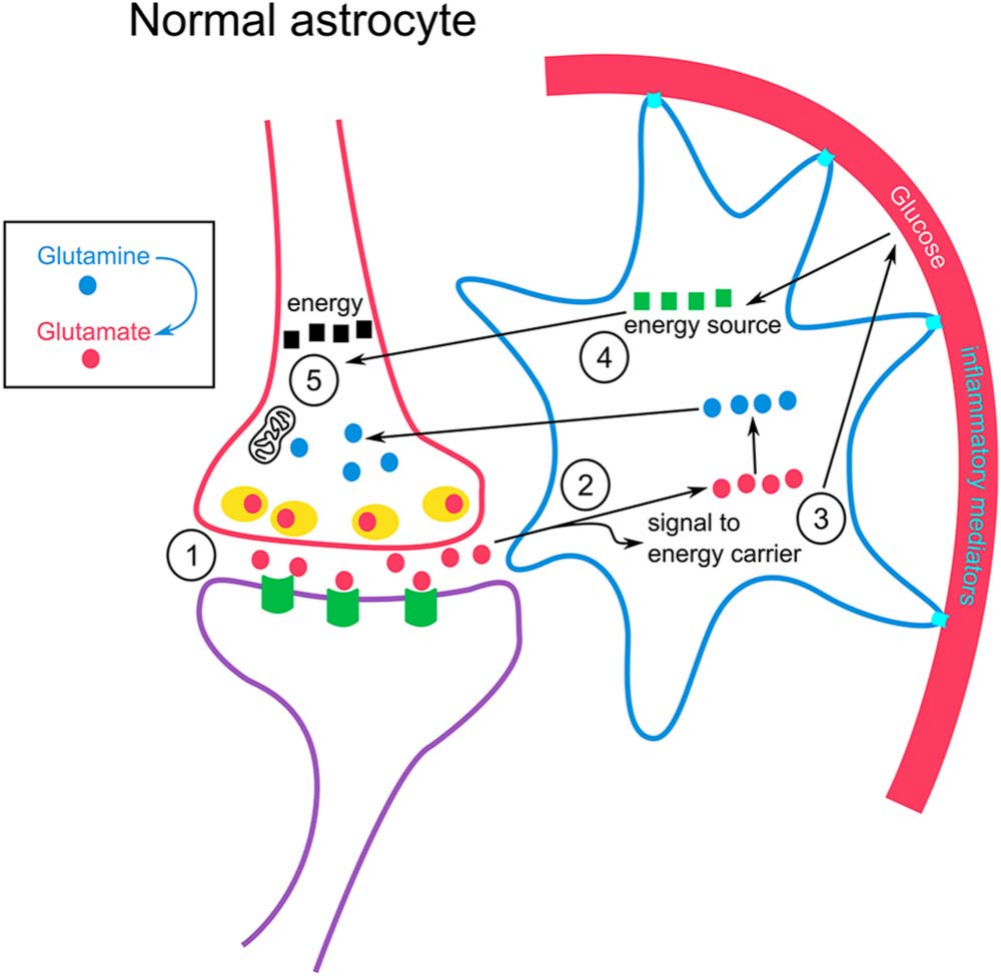
Glutamate handling by normal astrocytes. 1. Glutamate (red circles) release from presynaptic terminals and interaction with postsynaptic glutamate receptors (green symbols). 2. Uptake of glutamate into the astrocyte where glutamate is converted into glutamine (blue circles) and transported to presynaptic terminals where it is rebuilt into glutamate. 3. Glutamate in astrocytes signals to glucose carriers to take up energy source (green squares) from blood. 4. Glucose serves as an energy source for astrocytes and is transported to the neuron as lactate (black squares). 5. In neurons, lactate generates energy (ATP) via pyruvate from the mitochondria.

Glutamate signaling is complex and involves both neurons and astrocytes. Glutamate is synthesized in neurons and released from presynaptic terminals to activate postsynaptic receptors. After signaling, glutamate is taken up by astrocytes, whose processes surround synapses. Within astrocytes, glutamate is converted to glutamine, which is then transported back to neurons for resynthesis of glutamate and the inhibitory neurotransmitter gamma-aminobutyric acid (GABA). Concurrently, astrocytes signal to glucose transporters to increase glucose uptake from the bloodstream, thereby supplying additional energy to the brain (Fig. 1).

We suggest in our hypothesis that in conditions such as traumatic brain injury, stroke, CNS inflammation, or chronic stress, astrocytes become reactive, reducing their capacity to handle glutamate. This leads to:

- Reduced signaling across multiple neurotransmitter systems.
- Decreased energy supply to neurons.
- Nonspecific activation of larger neuronal circuits during information processing, due to extracellular glutamate accumulation from impaired astroglial uptake.

Interestingly, in reactive astrocytes, Ca^2+^ signaling is replaced by extracellular ATP signaling (64). ATP activates purinergic receptors on microglia and astrocytes, perpetuating an inflammatory environment and maintaining astrocyte reactivity (65).

## How can astrocytes be affected by autoimmunity?

We propose that in cases of systemic inflammation, such as autoimmunity, astrocytes are affected through impacts on the BBB. Mast cells and microglial cells appear to contribute to BBB disruption, allowing inflammatory mediators to enter the CNS. These mediators can further activate astrocytes, making them reactive and inflammatory, which reduces glutamate uptake. Collectively, these processes may provide a plausible explanation for brain fatigue in autoimmune diseases (see detailed references below).

## Astrocytes have strategic roles in the brain

Together with capillary endothelial cells, astrocyte processes regulate the BBB (58, 66, 67), the barrier that protects the CNS from soluble inflammatory mediators and effector immune cells. Astrocyte cell processes reach nerve cell bodies and envelop synapses (68). They are connected to each other in large networks through electric protein channels or gap junctions, through which low molecular weight substances and ions are transported (69, 70). In addition, astrocytes sense and are responsible for ion and amino acid homeostasis in the extracellular space and supply neurons with substrates for energy metabolism (71).

Brain cells require a constant and efficient energy supply, relying primarily on glucose delivered through the bloodstream for their highly specific functions. Cells need energy for ATP production; synthesis of neurotransmitters, such as acetylcholine, glutamate, and gamma-aminobutyric acid (71, 72, 73); and synaptic transmission (72). Glutamate is the main excitatory neurotransmitter (74) and central for information intake, processing, and storage of information in memory, and it is necessary for cognitive processes (75). Glutamate is released from the presynaptic terminal and affects receptors on the receiving (postsynaptic) nerve cells to create a signal within the neuronal cell networks (Fig. 1). Astrocytes clear the synapse cleft from glutamate as soon as it has exerted its effect using glutamate transporters. In this way, new signals can be sent with high specificity (76, 77, 78, 79, 80). On glutamate uptake, astroglial cell volume increases, primarily due to osmosis (76). Astrocytes and microglia synthesize and respond to pro-inflammatory cytokines. In case of damage or disease of the brain, these cells are activated and neuroinflammation occurs as protection (81).

## Astrocytes become inflammatory reactive

With increasing degree and duration of systemic inflammation, the BBB becomes more permeable to soluble factors and is infiltrated by innate immune cells (54). Autoimmunity in the CNS has a profound impact on astrocytes (81) and causes extracellular ATP signaling between the inflammatory activated astrocytes (64). Purinergic receptors on microglial, astroglial, and mast cells are activated (65, 82), thereby continuing the neuroinflammation and disruption of the BBB (83, 84).

**Figure 2:**
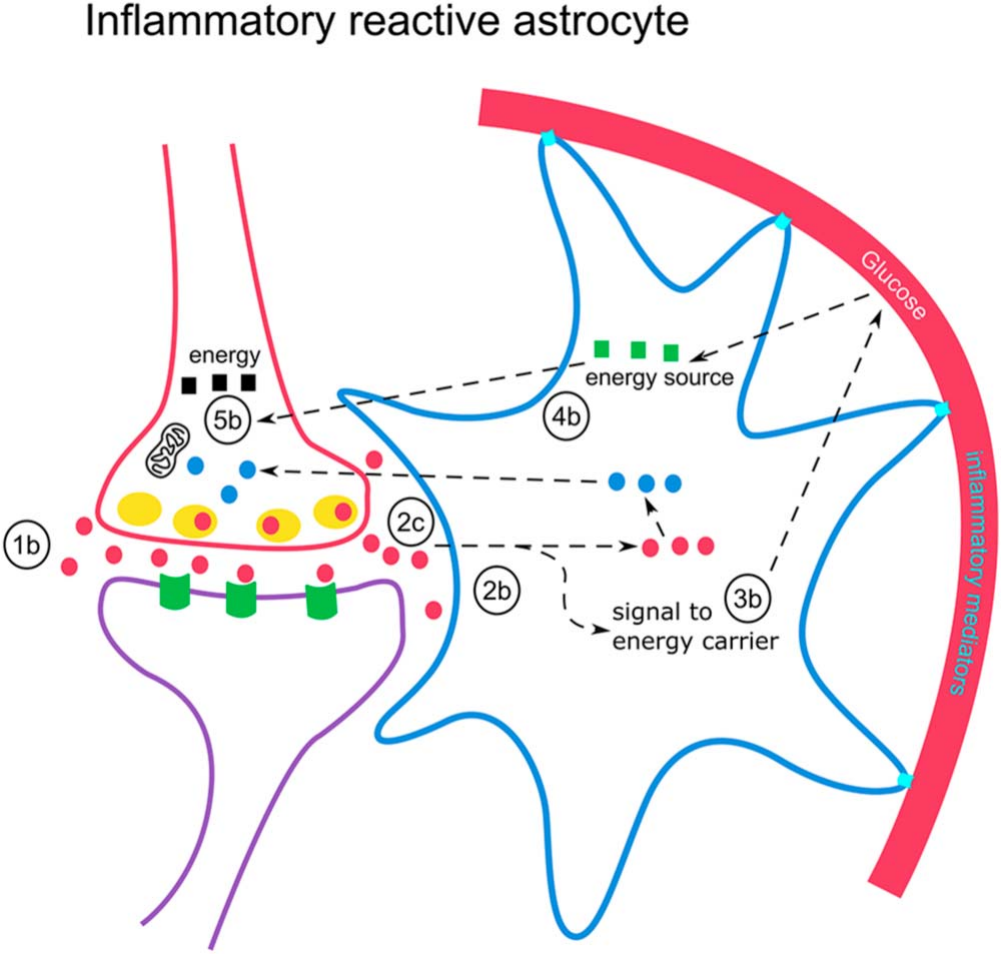
Impaired glutamate handling by inflammatory reactive astrocytes (see Fig. 1 for symbols). 2b. Impaired astrocyte glutamate uptake capacity. 2c. A slightly increased glutamate level in the extracellular space leads to more nonspecific information processing. 3b and 4b. An impaired energy supply from blood leads to less energy for astrocytes and neurons. 5b and 1b. An impaired glutamate transmission ultimately leads to impaired transmission for other neuronal signaling (dopamine, noradrenaline, serotonin, gamma-aminobutyric acid, and acetylcholine).

We know from studies of astroglial cells in cell cultures that, after injury or immune stimulation, their ability to effectively remove glutamate is affected (85) (Fig. 2). As a result, some glutamate remains in the synapse area after signaling has taken place, which in turn leads to less specific activation of the nerve cells. Another consequence is that less glutamate enters the astrocytes (81). This is problematic, as glutamate within the astrocytes signal to glucose carriers in the blood vessel walls to deliver glucose into the astrocyte (86). Glutamate in astrocytes is also used to manufacture glutamine, which is handed over to the nerve cells to produce new glutamate for continued signaling. Impairment of glutamate uptake by astrocytes could thus result in impaired uptake of glucose from the blood (87), and the increased extracellular glutamate may stimulate neuronal circuits nonspecifically (80).

Although much is still unknown, it appears that the hypometabolism and reduced glucose availability in many brain regions could account for reduced dopamine, norepinephrine, serotonin, and acetylcholine signaling (88). This could be of importance for associated symptoms within BFS.

## Hypothesis: dysfunction in astroglial support of glutamate transmission underlying brain fatigue in GD

In case of impaired astroglial fine-tuning of extracellular glutamate, glutamate signaling would be less specific (57, 76, 77, 89, 90). Due to impaired filter functions/suppression of irrelevant information, more information-bearing signals will be recognized as ‘new’ by sensory centers in the brain and reach the cerebral cortex for further handling. Therefore, larger neuronal circuits would be activated. An increase in extracellular glutamate could give rise to astrocyte swelling with a diminished extracellular space (91), and diffusion of extracellular glutamate to adjacent neurons activates these in a nonspecific way. As a result, more synapses would be activated and more astrocytes would be required to support this increased neuronal activity. An even more extended cell swelling will develop with significant disturbances in ion transport via the extracellular space (91). Impaired astroglial glutamate uptake decreases glucose uptake (77) and, consequently, the supply of metabolic substrates to neurons. The impaired astroglial handling of glutamate will also result in a decrease in glutamine supply to neurons and thereby decreased glutamate release from presynaptic terminals (Fig. 2). As referred to above, the relative hypometabolism may give rise to decreased signaling in other synaptic systems, which explains the appearance of the associated symptoms within brain fatigue. This may be indirectly reflected by current brain imaging data, which suggest altered brain function and connectivity associated with fatigue (92, 93, 94).

This hypothesis could explain why people with BFS may be able to perform cognitive tasks for short periods. In situations with heavy sensory stimulation, they soon feel exhausted, and it will take disproportionately long time to recover.

### How can brain fatigue symptoms persist in euthyroidism?

Why brain fatigue remains after treatment of GD in some patients is unclear. We have suggested a dysfunction in astroglial support of glutamate signaling as a hypothetical explanation, since inflammatory responses able to affect astroglia are activated in GD. The autoimmunity with systemic inflammation may induce neuroinflammation. In addition, astrocytes have been found to have extracellular ATP signaling in inflammatory and autoimmune conditions (95). Extracellular ATP can activate purinergic receptors on both astrocytes and microglial cells, which produce inflammatory mediators and thus maintain neuroinflammation (65, 96). This neuroinflammation may remain even after treatment of GD to euthyroidism, and the astrocyte dysfunction concerning glutamate handling may thus persist.

### Gaps in knowledge and ongoing research

It is not known whether the inflammatory activation in GD spreads to the CNS, but studies are ongoing according to ClinicalTrials.gov (clinicaltrials.gov: NCT05678374 and NCT06081439) to search for immunomarkers to detect BFS in blood and cerebrospinal fluid. Further research is needed to investigate whether there is a secondary neuroinflammation in GD due to penetration of the BBB by inflammatory mediators in the blood. With a knowledge-based approach, the glutamate transmission hypothesis (57) also needs to be tested in patients with GD. The brain effects from GD (seen also among hypothyroid patients) have been mainly studied by magnetic resonance imaging techniques, including functional magnetic resonance imagining, magnetic resonance spectroscopy, and single-photon emission computed tomography, but there are less data on biochemical and immunological markers mirroring processes in the CNS (12). Further research in this area is needed.

### Treatment for fatigue

It is essential that patients receive comprehensive information about fatigue and its potential impact on daily life. Temporary or partial sick leave is often necessary, particularly during the early stages of the disease, but may also be required for individuals experiencing persistent fatigue. Environmental adjustments, both at home and in the workplace, are essential, along with the implementation of energy conservation strategies. Support from an occupational therapist can be highly beneficial in this regard. Anxiety and depression frequently arise when daily routines are disrupted. Psychological support and treatment are crucial, and a psychologist can offer tailored interventions. Pharmacological and non-pharmacological treatments have shown promise in alleviating fatigue in patients with acquired brain injuries and multiple sclerosis. These include medications such as methylphenidate (62) and mindfulness-based therapies (97, 98, 99), but they have not been tested in GD, but studies are ongoing (clinicaltrials.gov: NCT06134219). Patient organizations can also play a valuable role in providing support and resources. However, the treatment of fatigue in GD remains an area requiring further investigation.

### Limitations of the hypothesis

As with many other psychiatric conditions, fatigue is difficult to test and quantify objectively, and both research and clinical assessment rely primarily on self-rating scales. Consequently, there is a need to validate fatigue in larger groups of patients with GD and to conduct further research to better understand and characterize BFS.

Our hypothesis is based on solid results from preclinical research, especially cell culture studies, over the past 3–4 decades. Research on the role of astrocytes in glutamate transmission was most prominent during the 1980s and 1990s. Results from more recent years have mostly confirmed previous research results. Most recently, there has also been an interest in pathological conditions, although with difficulties in studying humans.

## Conclusion

Fatigue is a common symptom among individuals with autoimmune diseases, including GD, and inflammation is suggested as a contributing factor. Our hypothesis is grounded in neurobiological knowledge and clinical experience with brain fatigue, developed by our group in conjunction with research on GD and other related conditions. However, this hypothesis requires further investigation in the context of GD, utilizing advanced methodologies such as metabolomics and other biochemical techniques. To fully understand the pathophysiology of brain fatigue in GD, studies employing human imaging technologies at the cellular level are likely necessary. Supporting patients in accepting and adapting to their limited energy resources is a crucial step toward improving their QoL. Equally important is the clinician’s role in recognizing fatigue and associated CNS dysfunction, which is essential for enhancing patient outcomes.

## Declaration of interest

HFN received lecture fees from Siemens, Orifarm, MSD, and IBSA; is a member of the professional council of the Swedish patient organization; and has led the work with the national task force of the national guideline of hyperthyroidism.

## Funding

This work was financed by grants from the Swedish state under the agreement between the Swedish government and the county councils, the ALF-agreement, the Wallenberg Center for Molecular and Translational Medicine, Västra Götaland Region, Gothenburg, Sweden, the Swedish Research Council, and the Novo Nordisk Foundation.

## Author contribution statement

All authors contributed to the manuscript and the development of the figures and approved the final version of the manuscript.
